# Face Protection for Children in Healthcare Settings

**DOI:** 10.3389/fped.2020.00553

**Published:** 2020-09-11

**Authors:** Vasiliki Vlacha, Gavriela Feketea

**Affiliations:** ^1^Department of Early Years Learning and Care, University of Ioannina, Ioannina, Greece; ^2^School “Iuliu Hatieganu” University of Medicine and Pharmacy, Cluj-Napoca, Romania; ^3^Department of Pediatrics, Pediatric Allergy Outpatient Clinic, “Karamandaneio” Children's Hospital, Patras, Greece

**Keywords:** children, COVID-19, face shield, pediatric primary care, personal protective equipment

The healthcare system in several countries is overwhelmed due to the COVID-19 pandemic. The ambulatory care settings carry a substantial patient burden with the risk of potential rise after the ending of the lockdown. The nosocomial transmission of SARS-CoV2 has been well-described (1). It is extremely important to minimize the viral transmission in the healthcare facilities among patients and the healthcare personnel as well (2). However, the implementation of safety measures becomes extremely challenging in ambulatory pediatric centers due to particular characteristics of children.

We would like to propose enhanced barrier precautions for pediatric patients of almost all ages as soon as they enter the ambulatory setting. These would include a face mask for children above 2 years of age and their care givers, according to CDC recommendation (3). Additionally, we also strongly recommend a face shield for children from 1 to 2 years of age. We also propose an alternative facial protective gear using a face shield for children aged between 2 and 5 years as a substitute for a face mask. These recommendations are based on our personal experience that toddlers resist wearing face masks and they tolerate face shields better. In addition, the face shield for children aged 1–2 years offers a face barrier without the risk of suffocation. The goal is to achieve the maximal protection for the pediatric patients of almost all ages and the healthcare workers.

The current suggestions are based on the unique characteristics of the pediatric population compared to adults. Most of the children, particular toddlers, cannot effectively practice social distancing so the use of a facepiece is required even more as a barrier to viral spread. Another marked characteristic is the crying behavior of children especially when they visit a medical setting. The dynamic of the SARS-Cov2 spread during crying has not been yet studied. However, the analysis of the peak expiratory airflow in premature newborns revealed that it was on average 6.6 times higher during crying than the flow during quiet breathing. Moreover, the ventilation during crying increased by 255% in comparison to quiet ventilation (4). Thus, crying probably facilitates the viral spread. Most importantly, the facepiece seems to control the transmission from asymptomatic carriers. It is well-known that the silent spreaders have high prevalence among children (5). Finally, the face protection equipment may eliminate the children's face-touching behavior. This could result in breaking the transmission by self-inoculation. The face shields as mode of protection against influenza virus have been shown to reduce the viral exposure by 96% in an 18-inch distance from a cough simulator (6). A systematic review analysis published by Chu summarizes the importance of social distancing and face/eye protection (7).

There are several limitations to our proposal. During the global shortage of personal protected equipment, the need of child size face masks and face shields would lead to exhaustion of manufacturing. In addition, some children could not tolerate wearing a facepiece even if repeatedly instructed to do so. A third issue could be the parental concern of potential suffocation by the face protectors requiring reassurance from the healthcare providers. We strongly advise the children to be under parental supervision while wearing a face-protective equipment.

Children's face shields are available in the market as sun-protective or anti-dust equipment, and some parents may be familiar with their usage. They come in different sizes according to the child's head circumference or they are adjustable. In Figure 1, you can see a handmade face shield made by sewing a transparent plastic sheet on a baby's hat. Written informed consent for publication of the child's figure was obtained from the child's mother.

**Figure 1:**
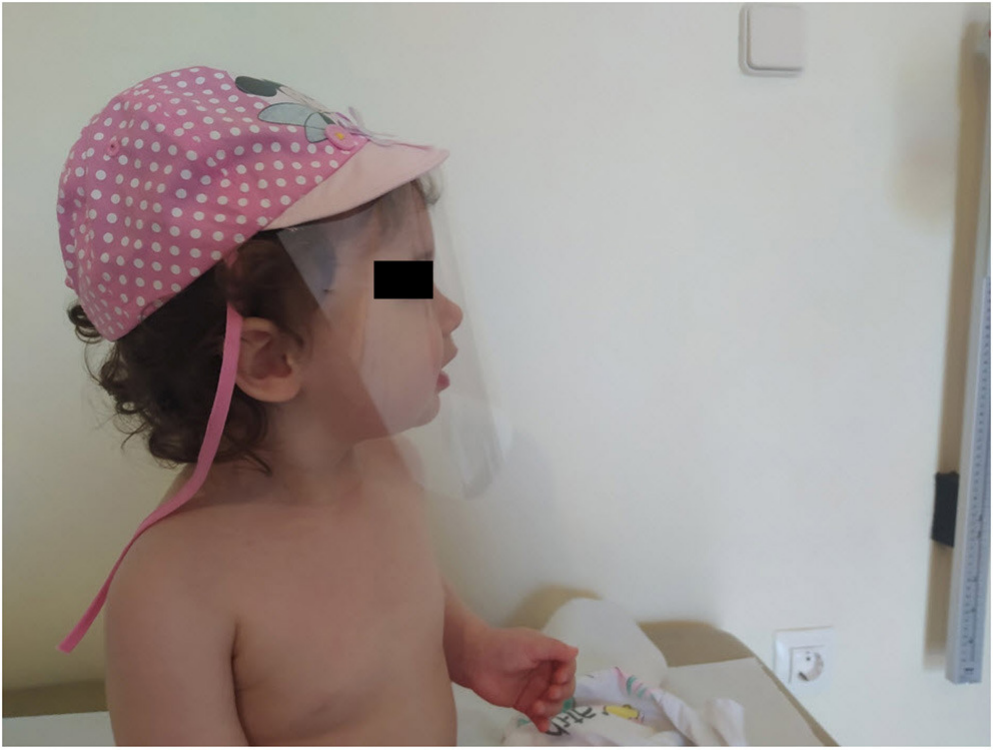
A 19-month-old child wearing a handmade face shield. It is being been published with permission.

It is important to apply additional safety measures for COVID-19 transmission, considering the unique children's characteristics, especially after lifting of pandemic restrictions.

## Author Contributions

VV and GF contributed to the design and implementation of the research, to the analysis of the results, and to the writing of the manuscript. All authors contributed to the article and approved the submitted version.

## Conflict of Interest

The authors declare that the research was conducted in the absence of any commercial or financial relationships that could be construed as a potential conflict of interest.
